# Diet-related selectivity of macroplastic ingestion in green turtles (*Chelonia mydas*) in the eastern Mediterranean

**DOI:** 10.1038/s41598-019-48086-4

**Published:** 2019-08-09

**Authors:** Emily M. Duncan, Jessica A. Arrowsmith, Charlotte E. Bain, Hannah Bowdery, Annette C. Broderick, Tierney Chalmers, Wayne J. Fuller, Tamara S. Galloway, Jonathon H. Lee, Penelope K. Lindeque, Lucy C. M. Omeyer, Robin T. E. Snape, Brendan J. Godley

**Affiliations:** 1 Marine Turtle Research Group, Centre for Ecology and Conservation, University of Exeter, Penryn, Cornwall TR10 9FE UK; 2 College of Life and Environmental Sciences: Biosciences, Geoffrey Pope Building, University of Exeter, Stocker Road, Exeter, EX4 4PY UK; 3 Marine Ecology and Biodiversity, Plymouth Marine Laboratory, Prospect Place, West Hoe, Plymouth, PL1 3DH UK; 4 Faculty of Veterinary Medicine, Near East University, Nicosia, North Cyprus Mersin 10 Turkey; 5 Society for Protection of Turtles, PK65, Kyrenia, North Cyprus Mersin 10 Turkey

**Keywords:** Ecology, Conservation biology

## Abstract

Understanding the drivers of key interactions between marine vertebrates and plastic pollution is now considered a research priority. Sea turtles are primarily visual predators, with the ability to discriminate according to colour and shape; therefore these factors play a role in feeding choices. Classification methodologies of ingested plastic currently do not record these variables, however here, refined protocols allow us to test the hypothesis that plastic is selectively ingested when it resembles the food items of green turtles (*Chelonia mydas*). Turtles in the eastern Mediterranean displayed strong diet-related selectivity towards certain types (sheet and threadlike), colours (black, clear and green) and shapes (linear items strongly preferred) of plastic when compared to the environmental baseline of plastic beach debris. There was a significant negative relationship between size of turtle (curved carapace length) and number/mass of plastic pieces ingested, which may be explained through naivety and/or ontogenetic shifts in diet. Further investigation in other species and sites are needed to more fully ascertain the role of selectivity in plastic ingestion in this marine vertebrate group.

## Introduction

The abundance and spatial distribution of plastic pollution in the world’s oceans is ever increasing, and thought to be emerging as one of the most ubiquitous and long-lasting changes in natural systems^1–3^. Extremely high densities of this pollutant are deposited along coastlines and in oceanic gyres^4,5^. Plastic debris enters the marine environment via a variety of pathways; the major source being terrestrial runoff (accounting for an estimated 80%) but additional sources include fisheries and maritime activities^6^.

The ingestion of plastic debris by marine vertebrates is now a global phenomenon. It is thought to occur in at least 43% of cetacean species, 36% of the seabird species, many species of fish and has been reported in all species of marine turtle^7–10^. Plastics are the most commonly ingested of all anthropogenic debris; with a wide variety of items found inside necropsied sea turtles^11–15^. This has the potential to cause lethal effects from intestinal blockage and injury but additionally adverse sub-lethal effects such as dietary dilution, malnutrition and impaired immunity^9^. Although debris ingestion in these species is considered a global research priority, the specific drivers and the levels of mortality caused are still poorly understood^16–18^.

When attempting to understand reasons for plastic ingestion it is important to consider the feeding ecology of marine turtles^11,15,19^. Consumption of plastic may be due to a failure of discrimination when mixed with normal dietary items. In juvenile green turtles (*Chelonia mydas*) in Brazil, plastic ingestion was thought to have occurred in conjunction with that of macroalagae due to debris entanglement in algal structures^20,21^. On the other hand, individuals may be actively selecting items, for instance, leatherback turtles (*Dermochelys coriacea*) are known to ingest plastic bags, presumably because they resemble their jellyfish prey^22^. Furthermore a high occurrence of plastic bottle lids ingested by loggerhead turtles (*Caretta caretta*) is thought to be because their round shape and presence floating near the surface means they resemble organisms that are normally preyed upon^12^. Such studies have prompted investigations into possible selective plastic ingestion^11^.

To promote an understanding of plastic ingestion in marine turtles, efforts have been extended towards documenting its prevalence. The EU Marine Strategy Framework Directive (2010) descriptor 10 included recommendations on future monitoring, suggesting loggerhead sea turtles would serve as a good indicator species to monitor the ecological quality within European waters if data on ingestion could be collected from stranded or bycaught specimens^14,23–25^. Building upon this, the Fulmar Protocol (*Fulmarus glacialis*; the indicator species for the North Sea)^26^ “toolkits” were created to unify methods for investigating plastic ingestion, allowing focus upon the differentiation between sources of ingested plastics^7,27,28^ i.e. the type of plastics ingested and their properties. Current suggested methodologies, however, neglect classifying other characteristics, such as colour and shape^28–30^. Sea turtles are primarily visual feeders and an ability to discriminate among colours and shapes has been shown to play a role in feeding choices^11,31^. Monitoring these aspects may offer insight into whether turtles are selectively ingesting some plastics. Data from beach plastic surveys have been used to set environmental baselines to investigate differences and selectivity with benthically feeding green and hawksbill (*Eretmochelys imbricata)* turtles (Queensland, Australia), which show a strong preference for ingesting clear sheet or rope like plastics and avoiding harder coloured pieces^11^.

Using data from stranded turtles we set out to test whether green turtles in the Eastern Mediterranean were selectively ingesting plastic that resembled their dietary items, typically seagrasses and algae^32^.

## Results

### Abundance of ingested plastic

**Figure 1:**
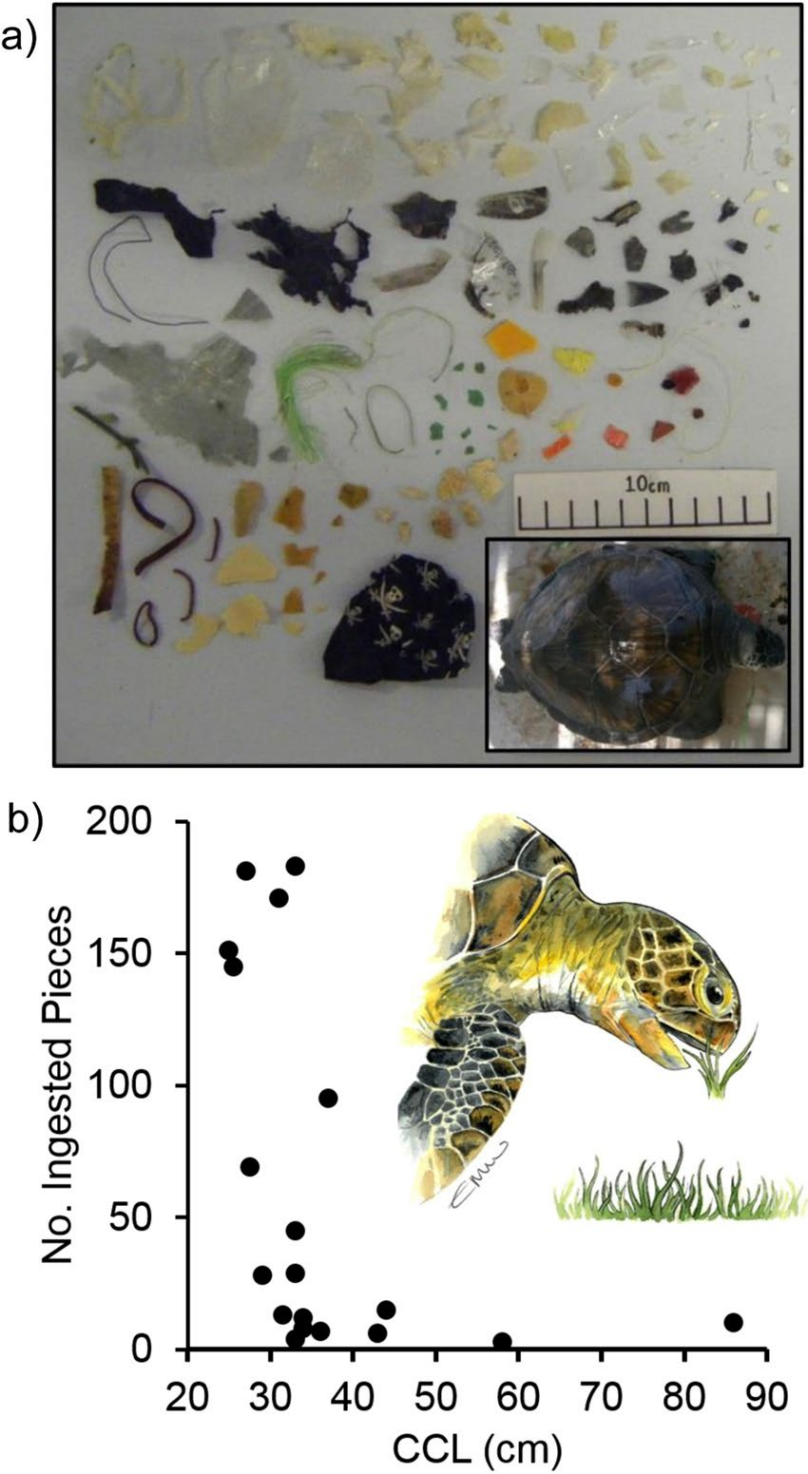
Macroplastic ingestion in green turtles (*Chelonia mydas*) from the Eastern Mediterranean. (**a**) Ingested plastic removed from the intestine of a juvenile (CCL = 33 cm; photo inset) showing the high quantities and diversity of plastic debris ingested. (**b**) Curved carapace length (cm) vs. the number of ingested pieces of plastic (n = 19). Original artwork by Emma Wood.

All green turtles, where whole GI tracts were available (n = 19), had ingested plastics with individuals having ingested an average of 61.8 ± 15.8 items (mean ± SE); ranging from 3–183 pieces (overall average weight 1.76 ± 0.53 g; ranging from 0.04–7.93 g) (Fig. 1a). The average mass of debris ingested per centimetre of turtle (converted SCL) was 0.065 ± 0.019 (mean ± SE; range 0.0007–0.266). Average body burden (g plastic/ kg turtle) was 0.585 ± 0.167 g/kg (mean ± SE; range 0.0009–1.970 g/kg). The majority of this plastic debris was found in the intestine section (100% occurrence) compared to the oesophagus (22%) or the stomach (33%) sections. For additional individuals (n = 15) for which stomach-only samples was available 27% contained ingested plastic.

There was a significant negative relationship between curved carapace length and the number (R_S_ = −0.658, n = 19, p = 0.002) (Fig. 1b) and mass of ingested plastic (g) (R_S_ = −0.592, n = 19, p = 0.008) with smaller animals generally having ingested more. This relationship was potentially powered by the values of two large individuals. However by removing these, the relationship between curved carapace length and the number of items still remained significant (R_S_ = −0.582, n = 17, p = 0.014) although that for mass of ingested plastic (g) became marginally non-significant (R_S_ = −0.431, n = 17, p = 0.083). With results normalised for body size there was also a strong significant negative relationship between body burden of plastic (g plastic/ kg turtle) and turtle size (converted SCL) (Rs = −0.781, n = 19, p < 0.001) as well turtle weight (converted kg) and number of ingested pieces (Rs = −0.657, n = 19, p = 0.002) or /and the number of ingested pieces per kg of turtle and turtle size (converted SCL) (Rs = −0.837, n = 19, p < 0.001) (Supporting Information Figs S3–S5). In addition there was a significant relationship between turtle body size (indicative of gape size of turtle) and mean length of ingested plastic (R_S_ = 0.553, n = 19, p = 0.014) but not mean area (2D) of ingested plastic (R_S_ = 0.219, n = 19, p = 0.369).

### Diet-related selectivity

**Figure 2:**
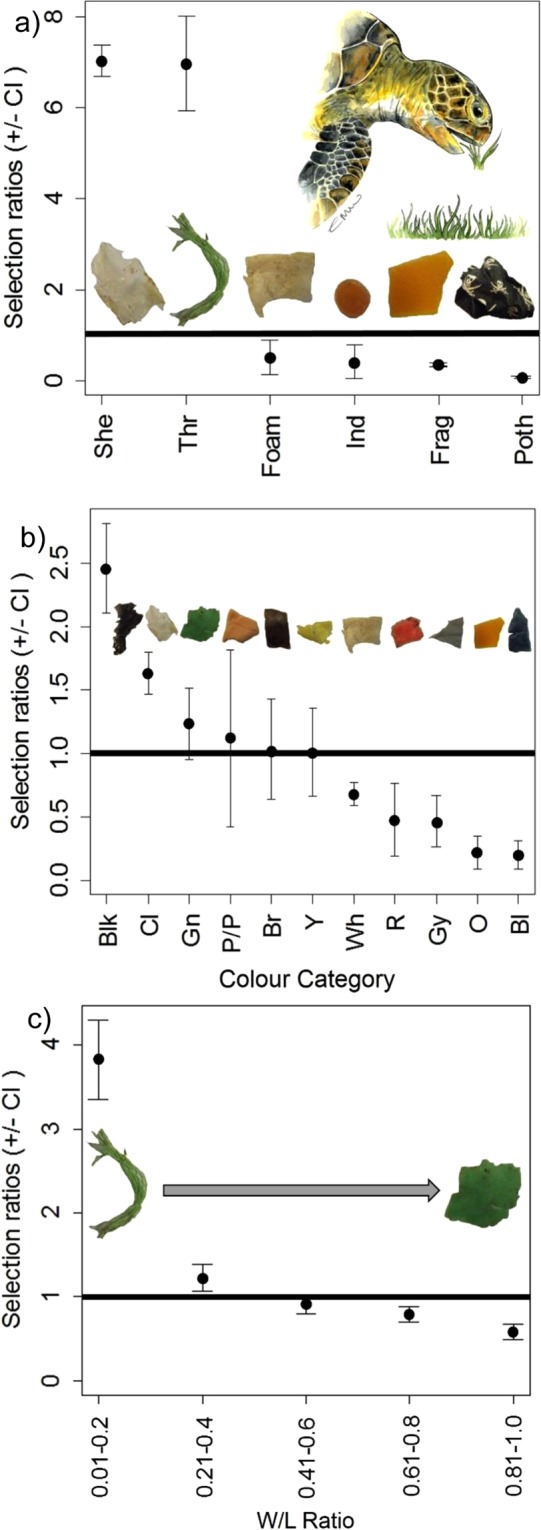
Marine turtle diet-related selectivity in macroplastic ingestion in the green turtles (*Chelonia mydas*) (n = 34). Manly’s Selectivity Ratios. A value > 1 this indicates a positive selectivity for that type/colour category than availability in the environment. Error bars indicate 95% confidence intervals. (**a**) type of plastic debris SHE = sheetlike plastics, THR = threadlike plastics, FOAM = foamed plastics, FRAG = hard plastics, POTH = other ‘plastic like’ items, IND = industrial nurdles (**b**) colour of plastic debris. Cl = Clear, Blk = Black, Y = Yellow, Wh = White, Gn = Green, Bl = Blue, Br = Brown, Gy = Grey, O = Orange, P/P = Pink/Purple, R = Red. (**c**) width/length ratio. If the ratio number produced was <0.2 this represented a rectangular shape whereas a ratio close to 1 indicated a more square or circular piece of debris. Original artwork by Emma Wood.

In relation to the ingested plastic, Manly’s selectivity ratio highlighted selectivity compared to environmental availability (Supporting Information Fig. S2). Calculated ratios showed green turtles exhibited a very strong selectivity towards both sheetlike and threadlike (wi = 7.033, wi = 6.968, respectively) plastic debris but appeared to avoid ingestion of foamed, hard fragments, “other pollutants” (e.g. rubber) and industrial types (Fig. 2a). When considering the ingestion of certain colour categories of plastic, the green turtles showed strong selectivity for *black*, *clear* and *green* debris (wi = 2.457, wi = 1.629, wi = 1.234, respectively) and also slight selectivity for *pink/purple*, *brown* and *yellow* debris while showing avoidance of *white*, *red*, *grey*, *orange* and *blue* plastics (Fig. 2b). In terms of debris shape, plastic with a small width:length ratio (long rectangular) were ingested at the highest frequency with turtles showing strong selectivity for lowest w/l ratios (wi = 3.823) and avoidance to higher w/l ratio values (more square or round) (Fig. 2c).

## Discussion

The current work suggests that green turtles (particularly juveniles) foraging in coastal waters of Cyprus regularly encounter and ingest plastic, so much so that the vast majority of animals contain some plastic in their GI tract, at the time of their death. Given the conservation status of this endangered species in the Mediterranean region^33^, the fact that consumed marine plastics are considered to have negative impacts and the high prevalence of plastics in this region, is an important finding^34^.

Alongside studies reporting predominance of seagrass in stomach contents, recent diet studies show that seagrass species such *Halophila stipulacea* and *Posidonia oceanica* contribute largely to the diet of neritic juveniles and almost exclusively to that of adult green turtles within this region (Palmer *et al*. unpublished data)^35,36^. Strong selectivity was exhibited by eastern Mediterranean green turtles towards plastics that potentially resemble their main dietary item, sea grass. Firstly, plastic types more preferably ingested were softer, more pliable plastics that tended to have a smaller width:length ratio therefore resembling these species of sea grass by shape and texture compared to our beach surveys and sea surface debris studies which is reported in the Mediterranean to be dominated by hard fragments of plastic^5^. Additionally the colours most selected for were *black*, *clear* and *green*, these colours more closely resemble sea grass in the water. Our findings indicates that turtles may not just be selecting plastics that look like gelatinous prey, which has been commonly stated in the literature as the “jellyfish hypothesis”, but other prey items.

Selective ingestion of plastic is plausible for green turtles as they have been shown to be capable of choosing particular species of seagrass over others or tending “grazing plots” therefore being selective in their natural feeding ecology^32^. Green turtles from Australia showed a strong preference for ingesting clear sheet or rope like plastics, avoiding harder, coloured (*blue*, *orange*, *red* and *yellow*) pieces which were foraging from coastal benthic habitats. The turtles in this part of Australia are thought to be herbivorous feeding principally on seagrass as well as a range of algae and mangrove fruits, with small immature turtles known to display selectivity in natural foraging habits; consuming plants with higher nitrogen and lower fibre levels^11,37^. This Australian study also included pelagic animals which were much less selective than their neritic counterparts; selectivity indices found not to be significantly different to environmental levels^11^.

Marine turtle visual biology and perception of colour could also greatly influence the ingestion of particular types or colours of debris^19^. Thayer’s law of countershading colouration in nature has been used to infer the likelihood of turtles detecting plastic fragments in the water column. Santos *et al*.^38^ suggested that marine animals that perceive floating plastic from below would preferentially ingest dark plastic fragments, whereas animals that perceive floating plastic from above would select for paler plastic. Our results for eastern Mediterranean green turtles are partially consistent with their study on Brazilian green turtles, with darker debris (black, green) ingested in greater proportions than available in the environment although it should be noted that clear pieces were also selected.

Finally, shape has been found to be an important factor in visual bycatch deterrents which have been shown to significantly reduce catch rate in green turtles, with the shape stimuli eliciting a response through visual physiology^39^. Alternatively, the shape of debris found in the GI tract could be influenced by biting behaviour, with turtles interacting with a large pieces of plastic and bitten off sections appearing during gut content analysis^40^.

It is important to note, however, that indirect ingestion of macroplastic through trophic transfer cannot be completely ruled out. Gelatinous macrozooplankton still make up a major component of the diet of neritic juvenile green turtles in the western Atlantic^15^. Macroplastic ingestion has been recently reported in such organisms providing potential trophic transfer of macroplastic pieces to marine turtles when prey is consumed suspended in the water column^41^. This is an unlikely as the turtles in this study, regardless of life stage, were largely feeding on seagrass. However dietary plasticity and their interest in items in the water column cannot be ruled out.

Size class or life history stage appears to be an important factor in determining the probability or variability of plastic ingestion as in previous studies on green turtles, despite the low sample size of larger turtles^15,42^. In addition g/cm and g/kg give a true indication of differential ingestion across the size classes of turtle as body size is normalized, with smaller turtles in this study having higher values for both units and therefore a higher body burden of ingested debris^10,13^. This may be a result of the feeding ecology and ontogenetic shifts in diet recognised in this species. During the early oceanic juvenile stage, turtles develop an epipelagic opportunistic feeding strategy, aggregating at frontal zones^43^, after which they typically recruit to neritic habitats and develop a more benthic herbivorous diet principally based on seagrass and algae^44^. In a Mediterranean assessment of dietary shift, seagrass prevailed in the stomach content of all turtles. The overall evidence (from gut content and stable isotope analysis) indicating a shift to a seagrass-based diet immediately after recruitment into neritic habitats^35^. Despite this, some could retain an omnivorous, less specialised diet for longer, which could explain variable ingestion of plastic debris within this life stage due to differences in the ontogenetic timing of diet specialisation^15,45–47^.

In both green and hawksbill turtles from Queensland, Australia, the probability of debris ingestion was inversely correlated with size, with smaller pelagic turtles significantly more likely to ingest debris and feed less selectively than larger benthic feeding turtles^11^. This might have other longer term consequences that could include reduced growth rates and therefore fecundity which could have long term demographic ramifications^3,9,12^. Future studies should aim to assess the impact on this particularly susceptible life stage.

To date, there have been relatively few studies within the Mediterranean on plastic ingestion by green turtles compared to current literature on the status of this threat in the loggerhead turtle population; where ingestion rates vary between 5–75%^7,25,27,28,48^. When comparing ingestion frequency occurrence (FO%) rates for the green turtles to those seen globally these are equivalent to some of the highest observed (in Brazil, others parts of South America and the central and North Pacific)^10,15,17,19,49,50^.

Currently the loggerhead turtle is the only indicator species for plastic ingestion in the Mediterranean for the Marine Strategy Framework Directive (GES Technical Subgroup on Marine Litter). However our results emphasise that green turtles are perhaps more likely to ingest plastic than loggerhead turtles in the same region. This highlights the importance of not simply focusing on a single indicator species to obtain a reliable indices of the impacts of this pollutant^23,28^.

Current methodological differences between studies limit comparison of the debris ingestion in sea turtles. There is no unified, globally used, classification system of ingested plastics in this group. Many recent studies focus upon the debris occurrence (%), however, factors potentially determining differences are overlooked, such as the characteristics of ingested plastic^51^. Indeed a recent review made a plea to quantify the amounts (g) of debris ingested and report these values these in terms of turtle size (e.g. g/kg, g/cm)^10^ as we have done here. The unification of plastic classification and the use of a singular categorisation method within the field would greatly aid intra- and inter- species comparisons and additionally comparisons with other taxa known to be affected by marine debris^14^. For example, the investigation of plastic ingestion in seabirds has benefited from the adoption of the Fulmar protocol, globally, with classification systems proving a cost effective biomonitor both in Europe and the North Pacific^52^. In addition, it should be noted that simply removing stomach contents to sample for macroplastic ingestion as initially suggested by Bjorndal *et al*.^53^ is not ideal as much of the retention of plastics occurs within the intestines, with the anterior portion of the rectum being shown to have the highest number of obstructions in this species^13,51^.

Following the work of Schuyler *et al*.^11^, beach plastic transects were used in this study as the only logistically feasible way to estimate environmental availability of plastic debris to green turtles. It is recognised there are some limitations to using this as a proxy due to differences in features influencing the resulting composition of plastic debris retained on beaches for example sheet-like material could be blown off the beach. Beach debris has, however, been widely used as the simplest and most cost- effective method to provide a reasonable proxy^20,42^. Items of beach debris are in a constant dynamic flux with the neritic marine environment, becoming re-suspended in the nearshore water^54^ and large volumes of data can be collected and characterised more comprehensively (n = 6106 items for this study) than possible from at-sea sampling. In the future, however, it is recommended that where in-water sampling is possible it should be used to provide quantitative estimates of plastic debris availability in conjunction with beach surveys. Furthermore future work on selectivity should also explore the possbiltiy of using a mutli-variate approach of anaylsis when larger sample sizes are available.

In conclusion, green turtles displayed strong diet-related selectivity towards certain types, colours and shapes of plastic when compared to the available environmental baseline, preferentially ingesting certain items even when they are likely less readily available in the environment. Colour and shape are factors that feed into the turtle’s foraging decision making. This study adds further support to the “active selectivity” hypothesis of plastic ingestion over the “accidental/ opportunistic” hypothesis that has also been proposed within the literature^11,20^. To understand the mechanisms of the “active selectivity” hypothesis, it is important to link this with known developmental biology and feeding ecology. Further species specific visual recordings would give greater insight into the selectivity of sea turtles in relation to ingested plastics based on a variety of physical properties^19^. Thus would lead to advances in this particular field and guide future research^21^.

## Materials and Methods

### Study area

This study was conducted on the island of Cyprus, in the Eastern Mediterranean basin. The island hosts important nesting beaches and foraging grounds for the Mediterranean population of green turtles (*Chelonia mydas*)^55^. The coastline is regularly patrolled for nest monitoring and stranded turtles, as well as having fisheries focused research and public awareness activities that led to the discovery, reporting and transportation of stranded or bycaught dead turtles to the author team for necropsy (with permission obtained from the North Cyprus Department of Environmental Protection). The vast majority of turtles subject to necropsy are considered to have resulted from bycatch incidents in coastal small-scale fisheries, typically being drowned in bottom-set trammel nets^56^. This adds to their suitability for the current study as they are likely representative of the population as a whole with regard to natural diet and plastic ingestion^10,51^.

### Necropsy and gut content analysis

During 2014–2016, green turtles with curved carapace length (min CCL i.e. notch to notch) ranging between 25 and 86 cm (36.9 ± 14.2 cm; mean ± SD; n = 19) were recovered dead and subject to necropsy (Supporting Information Fig. S1). The entire gastrointestinal tract was removed and subdivided into 3 parts: oesophagus, stomach and intestine. These sections of the gastrointestinal (GI) tract were analysed separately, initial contents were weighed and then rinsed through a 1 mm mesh sieve. After this, the remaining matter in the sieve was emptied into trays for sorting. Dietary items were separated, weighed and identified. Meanwhile suspected plastic or other marine debris was removed, cleaned and dried (to obtain dry mass) and stored for later analysis. To normalize for turtle size we calculated g plastic/cm of turtle and g plastic / kg turtle following calculations outlined in Clukey *et al*.^13^ and Lynch^10,57^. For selectivity analysis, data were augmented with stomach-only samples from green turtles from sample years 2011–2013 (n = 15; similar body size distribution) to allow for a larger sample of ingested debris when focusing on the physical properties.

### Novel plastic classification methodology

The novel classification used in this study builds upon the Fulmar Protocol and MSFD (Marine Strategy Framework Directive) Marine Litter Report 2011 (Descriptor 10) “toolkits”. This involves categorising plastic debris type into the following: Industrial plastic pellets or nurdles (IND) and user plastics (USE) which can be split into several sub-categories; sheetlike plastics (SHE) e.g. plastic bags, threadlike plastic (THR) e.g. remains of rope, foamed plastics (FOAM) e.g. polystyrene, fragments (FRAG) e.g. hard plastic items and other (POTH) e.g. rubber, elastics, items that are ‘plastic-like’ that do not clearly fit into another category. Dry weight was (mg) taken of every individual piece isolated^26^. Additional recordings of colour and three dimensional measurements (length, width and depth) of each individual piece of plastic were also taken. Colour was recorded within 11 categories; Clear, White, Pink/Purple, Red, Orange, Yellow, Green, Blue, Brown, Black, Grey. Width: Length ratios were calculated (W/L). A ratio close to 1 indicated a square or round 2D piece of debris with ratios < 1 leading to rectangular and progressively more linear shapes with decreasing ratio. Area was calculated on a 2D plane (L*W).

To gain an environmental baseline, 17 beaches distributed around the coastline were sampled between July-August 2016 for deposited plastic marine debris (see Supporting Information). Beach survey is regarded as the simplest and most cost- effective method to provide a reasonable proxy for marine debris environmental availability^58^. The beaches of N Cyprus display a high burden of marine source and low land-based input of plastic debris due to the current and wind patterns around the coastline in addition to low population and human visitation to the beaches^59^. Cyprus is situated in the Levantine Basin with minimal interaction with the western Mediterranean. Hydrodynamic (current) models illustrate the anticlockwise currents in this basin. The coastline receives debris from offshore accumulation zones due to the Shikmona anticyclone gyre (SMA) off the SE coast of Cyprus, this plastic is then caught in strong north-eastern currents and carried up the E coast Cyprus and deposited along the windward N coast. Beach plastic items (n = 6106) were subject to the same measurements as those pieces ingested by turtles.

### Statistical analysis

We calculated Manly’s selectivity ratio for debris type, colour and shape. In the past this method has been used widely to estimate for habitat or diet selection but more recently has been used to explore the selectivity of plastic debris because the index takes into account the availability of each debris type and colour in the environment^11^. If the value calculated is >1 this indicates a positive selectivity for that type/colour category, suggesting that turtles target that type of plastic compared to what is available in the environment. However a value <1 indicates a negative selectivity to that category, suggesting avoidance of that debris type in the environment.

## Supplementary information

Supplemental Material

## Data Availability

The datasets generated during and/or analysed during the current study are available from the corresponding author on request.
