# Supplementary material for: Diet-related selectivity of macroplastic ingestion in green turtles (Chelonia mydas) in the eastern Mediterranean
Source: Sci Rep. 2019 Aug 9;9:11581. doi: 10.1038/s41598-019-48086-4 (PMC6688982; doi:10.1038/s41598-019-48086-4)
Supplement: Supplementary file 1 — Supplemental Material [file 41598_2019_48086_MOESM1_ESM.docx]

**Title:** Diet-related selectivity of macroplastic ingestion in green turtles (*Chelonia mydas*) in the eastern Mediterranean

Emily M. Duncan ^1, 2, 3^, Jessica A. Arrowsmith^1^ ,Charlotte E. Bain^1^, Hannah Bowdery^1^ , Annette C. Broderick^1^, Tierney Chalmers^1^,Wayne J. Fuller ^1,4,5^,Tamara S. Galloway^2^ , Jonathon H. Lee^1^,Penelope K. Lindeque^3^,Lucy C. M. Omeyer ^1^,Robin T. Snape ^1,5^, Brendan J. Godley*^1^

^1^ Marine Turtle Research Group, Centre for Ecology and Conservation, University of Exeter, Penryn, Cornwall, UK, TR10 9FE

^2^ College of Life and Environmental Sciences: Biosciences, Geoffrey Pope Building, University of Exeter, Stocker Road, Exeter, UK, EX4 4PY

^3^Marine Ecology and Biodiversity, Plymouth Marine Laboratory, Prospect Place, West Hoe, Plymouth, UK, PL1 3DH

^4^Faculty of Veterinary Medicine, Near East University, Nicosia, North Cyprus Mersin 10, Turkey

^5^Society for Protection of Turtles, PK65, Kyrenia, North Cyprus Mersin 10, Turkey

**Supporting Information**

**Beach surveys**

**Study Area**

Sampling was carried out at 17 beaches along the north and east coast of Cyprus, Eastern Mediterranean between July and August 2016. Surveys were organised to coincide with the main period of turtle nesting/hatching activity. Beaches were selected, based upon their spatial distribution and high turtle nesting densities.

**Sediment Sampling**

Within each beach, data were collected from 10 pairs of sampling sites along two lines parallel to the shore: the ‘strandline’ and “transect of typical turtle nesting area”. Strandline (SL) was defined as the highest line of debris left from the retreating tide. This meandering line where debris accumulates is periodically generated by tide and exposed air movements (Heo et al. 2013); the transect through turtle nesting area was approximately the median distance between strandline and the landward limit of the beach within which turtles nested, approximated by a) marked nests, b) body pits left from nesting attempts.

The 10 sampling sites were spaced equidistantly, with sample 1 and 10 lying 5% of the beach length from each end to avoid rocky edges of the beach.

All samples were collected using a cylindrical metal corer of 20cm diameter and 60cm height. All sand and debris was gathered for 0-2cm depth at sampling locations on the strandline and the nesting area. At locations in the nesting area a volume of 250cm^3^ was taken from incremental depths (2.1-10.0cm, 10.1-20.0, 20.1-30.0, 30.1-40.0, 40.1-50.0, 50.1-60.0cm) unless water or rock was struck first. Each subsample was air dried in metal trays before being sieved.

**Plastic Separation & Categorisation**

Anthropogenic waste of 5-200mm sizing was gathered from the top mesh (5mm) defined as macroplastic (>5mm), the size class used as the environmental baseline to this study. Plastic debris were removed by eye to be analysed and categorised by the classification method stated as set out by the Fulmar Protocol and MSFD (Marine Strategy Framework Directive) Marine Litter Report 2011 (Descriptor 10) “toolkits” including type and colour of plastics (n=6106). To gain a baseline for shape and size of plastics this dataset was augmented with further beach surveys during August 2017 (n=1167). There was no significant difference between the original and augmented data set in terms of type (Wilcoxon rank sum test:W=16.5, p=0.463) or colour (W=84.5,p=0.123) of plastic debris in samples.


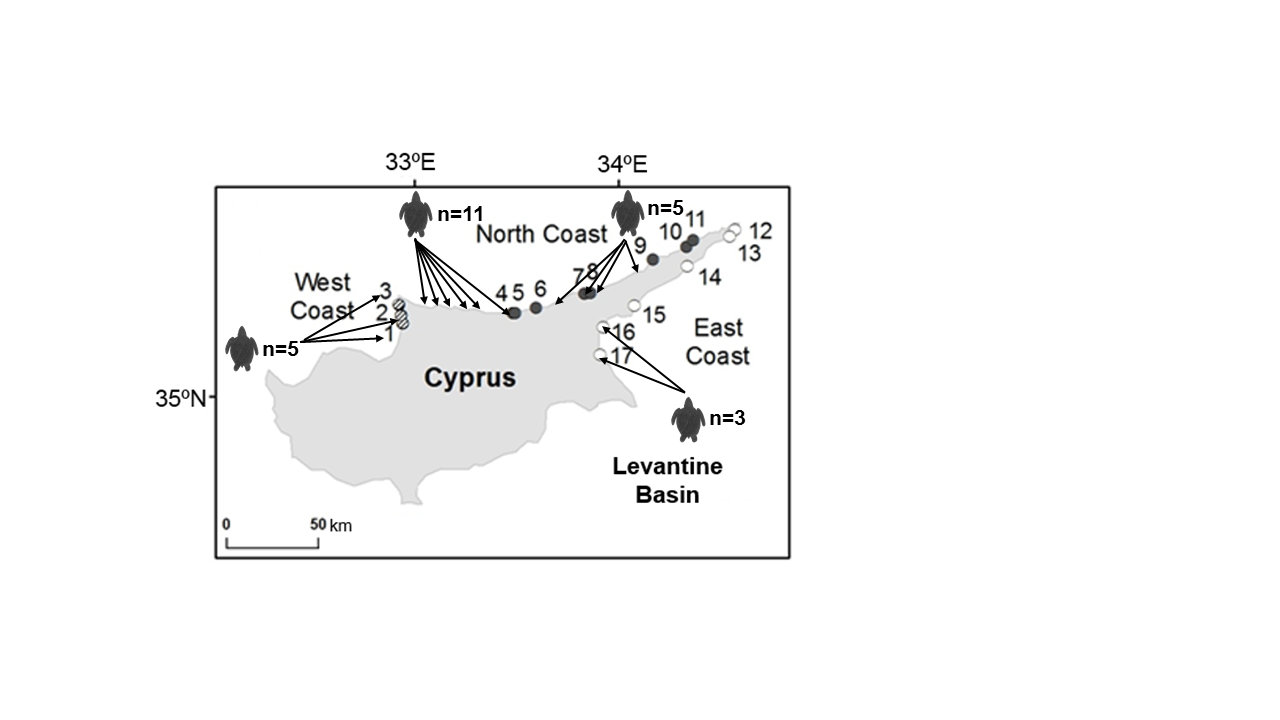


**Figure S1. Map of stranding locations and beach debris sampling sites on the coast Cyprus.** Stranding locations (n=24) for turtles with stranding sites recorded. Beach debris sampling sites (n=17).


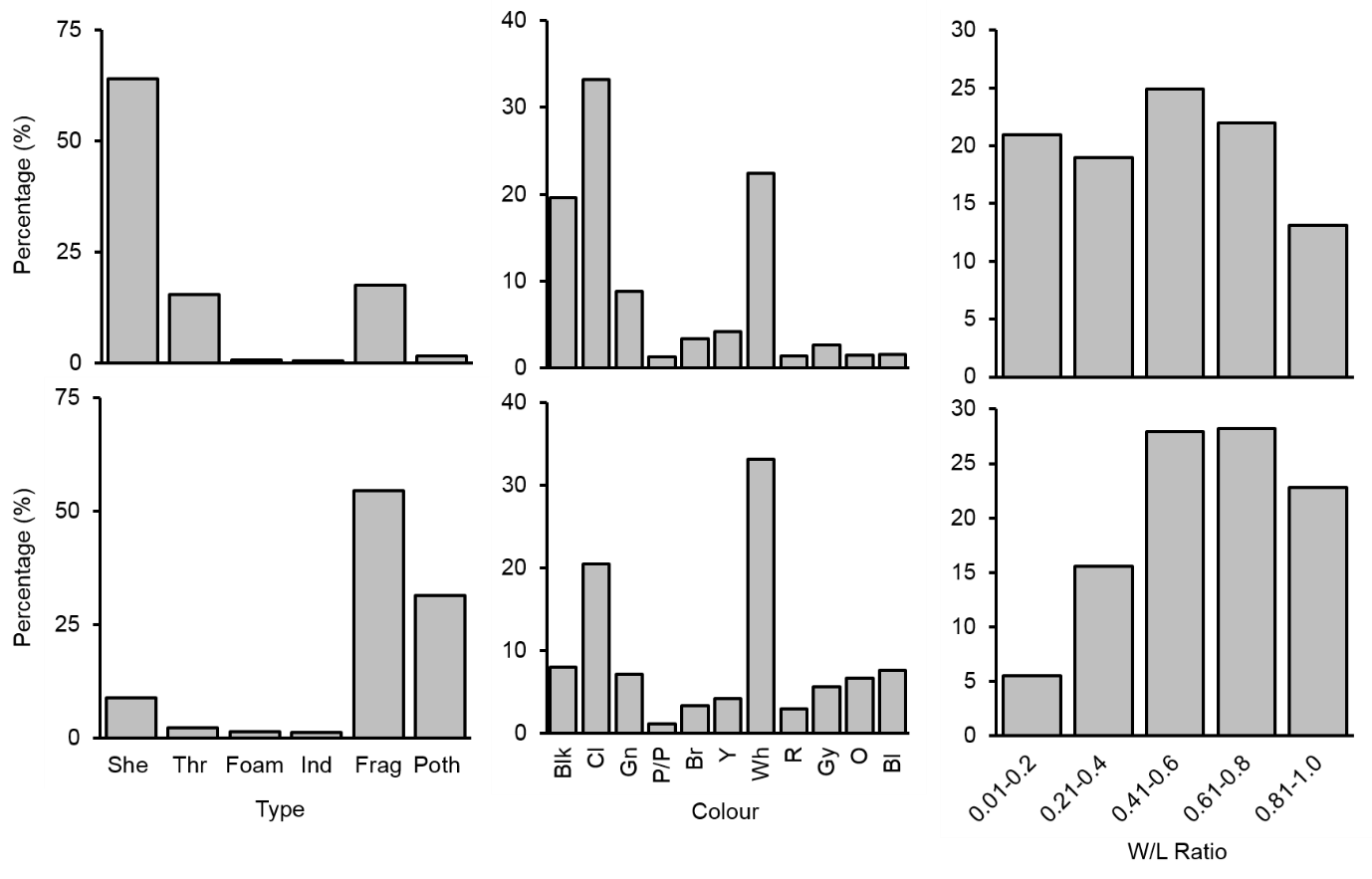


f)

e)

d)

c)

b)

a)

**Figure S2. Type, colour and size of plastic debris comparison ingested by marine turtles and beach surveys.** Type of a) ingested and b) beach plastic debris; SHE=sheetlike plastics, THR=threadlike plastics, FOAM= foamed plastics, FRAG= hard plastics, POTH= other ‘plastic like’ items, IND= industrial nurdles (n=6106). Colour of a) ingested and b) beach plastic debris. Cl=Clear, Blk=Black, Y=Yellow, Wh=White, Gn=Green, Bl=Blue, Br=Brown, Gy=Grey, O=Orange, P/P=Pink/Purple, R=Red (n=4269). Width/length ratio e) ingested and f) beach plastic debris. If the ratio number produced was <0.2 this represented a rectangular shape whereas a ratio close to 1 indicated a more square or circular piece of debris (n=1167)

**Figure S3.** Body burden (g plastic/kg turtle) in relation to turtle straight carapace length (SCL) (n=19)

**Figure S4.** The number of ingested pieces of plastic vs. turtle weight (kg) (n=19).

**Figure S5.** Number of ingested pieces per kg of turtle in relation to turtle straight carapace length (SCL) (n=19)


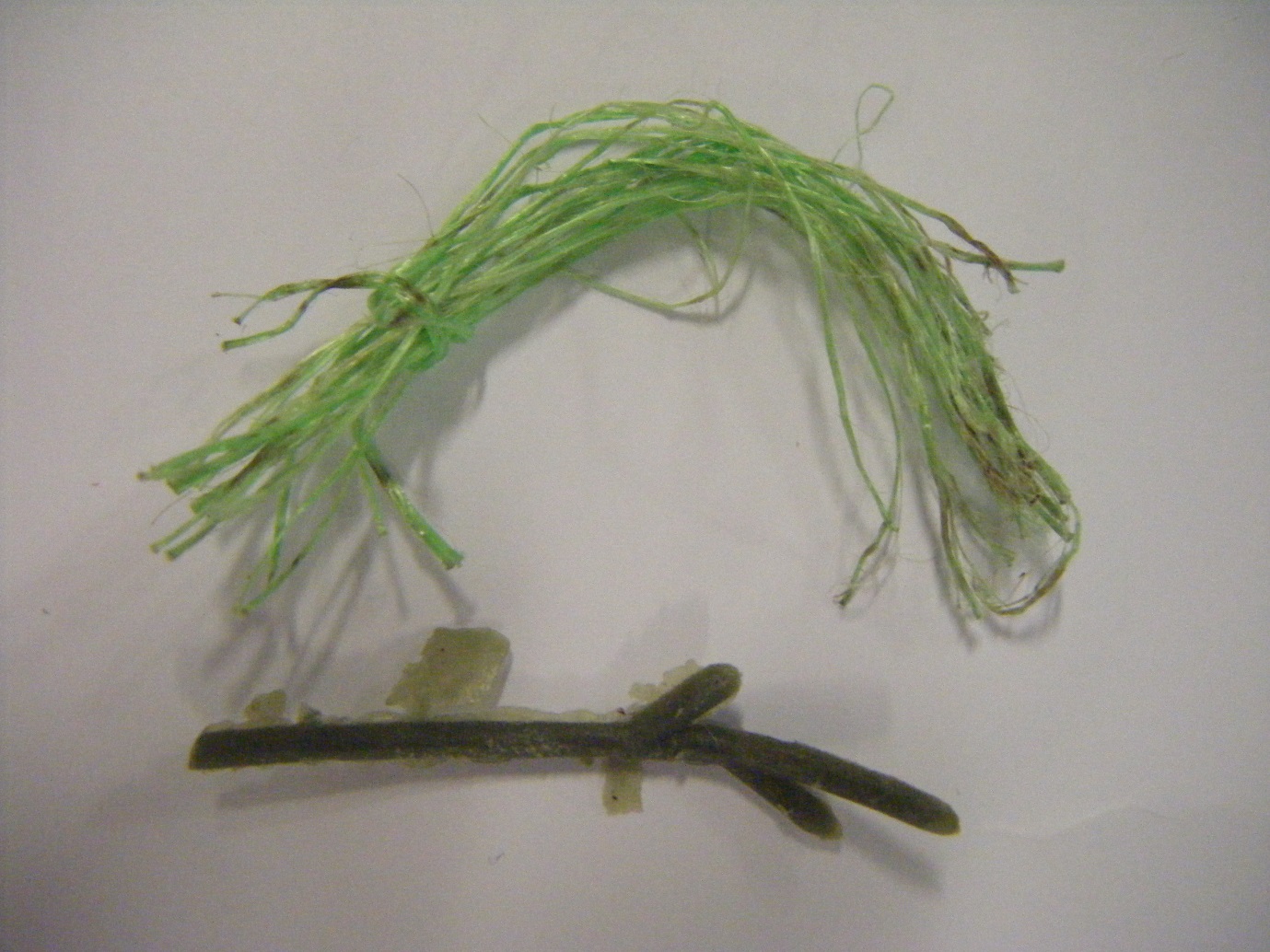


**Figure S6. Examples of ingested plastic by green marine turtles;** type, colour and size showing similarities to dietary items such as seagrass
